# Indel‐seq: a fast‐forward genetics approach for identification of trait‐associated putative candidate genomic regions and its application in pigeonpea (*Cajanus cajan*)

**DOI:** 10.1111/pbi.12685

**Published:** 2017-02-09

**Authors:** Vikas K. Singh, Aamir W. Khan, Rachit K. Saxena, Pallavi Sinha, Sandip M. Kale, Swathi Parupalli, Vinay Kumar, Annapurna Chitikineni, Suryanarayana Vechalapu, Chanda Venkata Sameer Kumar, Mamta Sharma, Anuradha Ghanta, Kalinati Narasimhan Yamini, Sonnappa Muniswamy, Rajeev K. Varshney

**Affiliations:** ^1^ International Crops Research Institute for the Semi‐Arid Tropics Patancheru Telangana State India; ^2^ Agricultural Research Station (ARS)‐Tandur Professor Jayashankar Telangana State Agricultural University (PJTSAU) Hyderabad Telangana State India; ^3^ Agricultural Research Station (ARS)‐Gulbarga University of Agricultural Sciences (UAS) Raichur Karnataka India; ^4^ School of Plant Biology and Institute of Agriculture The University of Western Australia Crawley WA Australia

**Keywords:** bulked segregant analysis, fusarium wilt, Indels, sterility mosaic disease, whole‐genome resequencing

## Abstract

Identification of candidate genomic regions associated with target traits using conventional mapping methods is challenging and time‐consuming. In recent years, a number of single nucleotide polymorphism (SNP)‐based mapping approaches have been developed and used for identification of candidate/putative genomic regions. However, in the majority of these studies, insertion–deletion (Indel) were largely ignored. For efficient use of Indels in mapping target traits, we propose Indel‐seq approach, which is a combination of whole‐genome resequencing (WGRS) and bulked segregant analysis (BSA) and relies on the Indel frequencies in extreme bulks. Deployment of Indel‐seq approach for identification of candidate genomic regions associated with fusarium wilt (FW) and sterility mosaic disease (SMD) resistance in pigeonpea has identified 16 Indels affecting 26 putative candidate genes. Of these 26 affected putative candidate genes, 24 genes showed effect in the upstream/downstream of the genic region and two genes showed effect in the genes. Validation of these 16 candidate Indels in other FW‐ and SMD‐resistant and FW‐ and SMD‐susceptible genotypes revealed a significant association of five Indels (three for FW and two for SMD resistance). Comparative analysis of Indel‐seq with other genetic mapping approaches highlighted the importance of the approach in identification of significant genomic regions associated with target traits. Therefore, the Indel‐seq approach can be used for quick and precise identification of candidate genomic regions for any target traits in any crop species.

## Introduction

Conventional trait mapping methods are generally expensive and take much time in generating and analysing genotyping data on segregating populations. Trait mapping becomes more time‐consuming if genotyping is performed using low‐throughput marker systems such as simple sequence repeat (SSR) markers. Visual scoring in such marker systems also adds to the possibility of discovering spurious marker–trait associations (MTAs). High‐throughput marker systems such as single nucleotide polymorphism (SNP) in combination with automated genotyping platforms (SNP arrays, KASpar assays, GoldenGate assays, etc.) have provided better options in generation of genotyping data. However, downstream analysis of such large volume data (quality assessment, identification of parental polymorphism and subsequently assessment of informative SNPs in population) takes time to provide meaningful information, which can be used for MTAs. This limits the rapid deployment of high probability MTAs in genomics‐assisted breeding (GAB) and, subsequently, delays development of new breeding lines (Varshney *et al*., 2007). Additionally, meeting the increasing demand of nutritious food under anticipated climate change scenario along with ever‐decreasing agricultural lands and limited water resources is a challenging task (Khoury *et al*., 2014). It requires sophisticated rapid genome mapping and targeted GAB approaches to produce better and high‐yielding crop varieties in faster manner (Godfray, 2010; Varshney *et al*., 2005).

The rapid development of next‐generation sequencing (NGS) technologies has enabled generation of genomic resources at large scale with faster pace during the last decade (Pazhamala *et al*., 2015; Pandey *et al*., 2016). NGS‐based approaches have also provided rapid ways to establish relationship between genotype and phenotype at higher resolution (Varshney *et al*., 2014). Nevertheless, despite the decreasing sequencing cost, development of individual reference‐based assembly for each accession in a given species or progeny of mapping populations is still a challenging task. To overcome this bottleneck and to identify genomic segments responsible for phenotypic traits using NGS, many alternative approaches such as SHOREmap (Schneeberger *et al*., 2009), Next‐generation mapping (NGM) (Austin *et al*., 2011), MutMap (Abe *et al*., 2012), Isogenic mapping‐by‐sequencing (Hartwig *et al*., 2012), SNP‐ratio mapping (SRM) (Lindner *et al*., 2012), MutMap+ (Fekih *et al*., 2013), MutMap‐Gap (Takagi *et al*., 2013a) have been used. Above‐mentioned studies rely on a number of different principles, which can handle mainly qualitative traits (traits governed by 1‐2 genes). In contrast, QTL‐seq approach was proposed primarily to deal with quantitative traits, based on Δ SNP index to map the target genomic region(s) for blast resistance and seedling vigour in rice (Takagi *et al*., 2013b). Similarly, whole‐genome resequencing (WGRS)‐based BSA was applied to calculate G′ statistics to identify the QTLs for cold tolerance in rice seedling (Yang *et al*., 2013). Recently, genome resequencing of contrasting parents together with identification of nonsynonymous SNP (nsSNP) substitution was utilized for identification of candidate genes in defined QTL regions or new genic regions in many crops (Silva *et al*., 2012; Singh *et al*., 2016a; Xu *et al*., 2014). To list a few, nsSNP substitution approach has been successfully utilized in mapping the candidate genes for sheath blight resistance in rice (Silva *et al*., 2012), drought tolerance in maize (Xu *et al*., 2014) fusarium wilt (FW) and sterility mosaic disease (SMD) resistance in pigeonpea (Singh *et al*., 2016a). In all these studies, SNP genotyping data were used for establishing MTAs. However, Indels in the genomic regions based on bulked segregant sequencing have not yet been targeted for trait mapping. Evidence of involvement of Indels in altering the gene functions has been reported in different crops (see Kage et al., 2015). Further, in comparison with other markers, Indels have a number of inherent advantages such as abundance in the genome, multi‐allelic and codominant, ease in marker conversion and amenable to low‐cost genotyping.

In view of above, this study reports a novel approach called ‘Indel‐seq’, which is a combination of WGRS and BSA, for the identification of Indels associated with target traits. An example of application of Indel‐seq has been provided in pigeonpea with FW and SMD resistance as target traits. In this context, the extreme bulks (resistant and susceptible) along with the resistant parents of recombinant inbred lines (RILs) segregating for FW and SMD resistance were sequenced. Candidate genomic regions/genes were identified for FW and SMD resistance in pigeonpea using Indel‐seq approach. Further, the identified Indels were validated on a set of FW‐ and SMD‐resistant and FW‐ and SMD‐susceptible genotypes. In summary, Indel‐seq seems to be a suitable approach for coarse as well as fine mapping of quantitative traits in a rapid and precise manner.

## Results

### Principle of Indel‐seq

**Figure 1:**
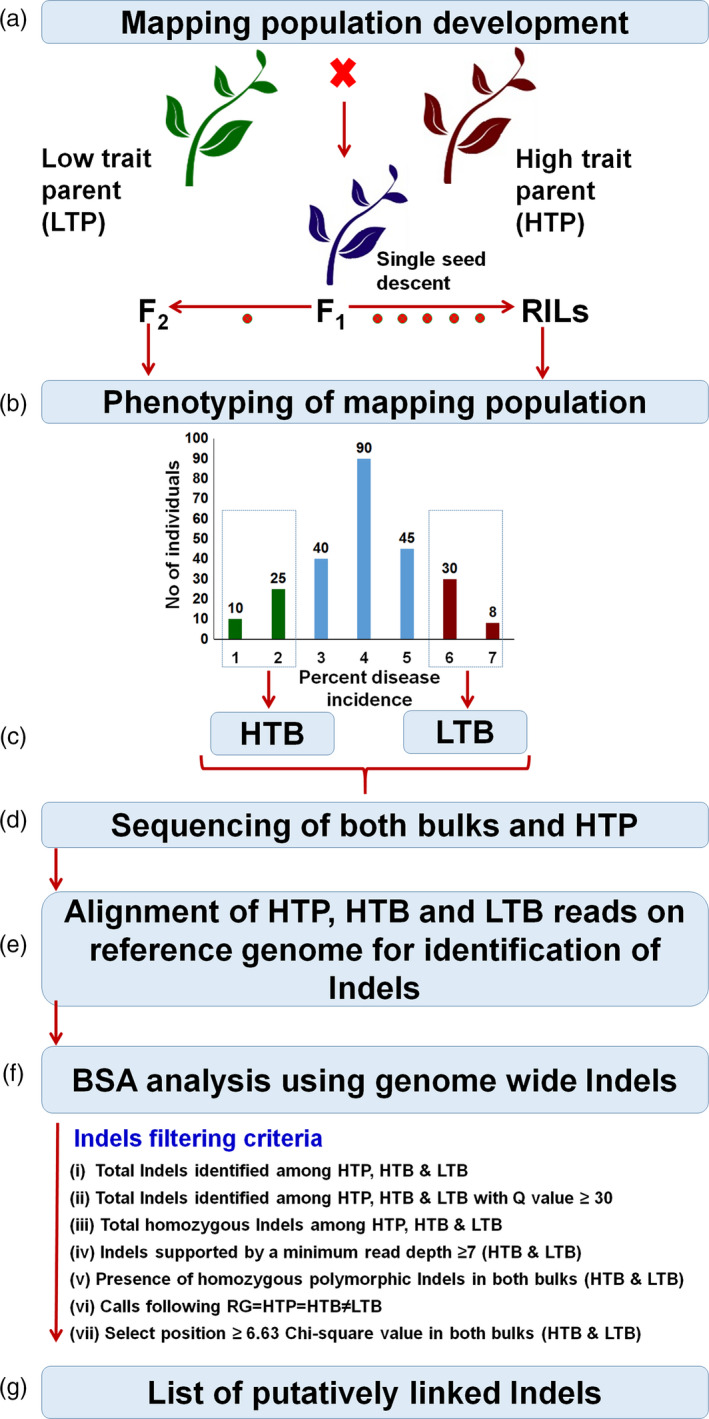
Pipeline of Indel‐seq approach. (a) Two contrasting parents (high trait parent (HTP) and low trait parent (LTP) are crossed to develop segregating population (F_2_/RILs) for target traits). (b) Based on the phenotyping of mapping population for the target traits, ~20 plants with extreme phenotype are selected for the constitution of extreme pools. (c) Low trait bulk (LTB) and high trait bulk (HTB) are constructed based on equimolar bulking of ~20 individuals of DNA for each bulk. (d) These two DNA bulks along with high trait parent (HTP) are used to whole‐genome resequencing. (e) Raw reads of HTP, HTB and LTB are aligned to the reference genome (RG, which is similar to high trait parent in term of target phenotype) for the identification of Indels. (f) Bulked segregant analysis (BSA) approach is applied to identify the associated Indels with the target traits using several Indel filtering criteria to identify putatively associated Indels between resistance and susceptibility. (g) List of putatively linked Indels identified.

Indel‐seq combines WGRS and BSA to identify the genomic regions associated with the target traits. To initiate Indel‐seq approach, any segregating population (F_2_/RILs/back‐cross) for the target traits could be utilized. Based on the phenotypic data of segregating population, 15–20 lines of extreme classes can be selected to constitute DNA pools in high trait bulk (HTB) and low trait bulk (LTB). Subsequently, two bulks (HTB and LTB) along with the high trait parent (HTP) are subjected to WGRS with high genome coverage (~10×) (Figure 1). WGRS data, subsequently, can be analysed in a proposed manner to detect trait(s)‐associated Indels. As the first step in analysis, high‐quality WGRS data from HTP, HTB and LTB are mapped to the reference genome (RG). Mapped/aligned data are used for the identification of genomewide Indels. Identified Indels are then subjected to high‐quality filtering parameters such as Q value >30, homozygous and no ‘N’ (missing call) in any tested sample. Further homozygous Indels supported by a minimum of seven sequencing reads in both the bulks (HTB and LTB) can be selected for establishing MTAs. In this direction, each Indel could be passed through the either (i) or (ii) of following criteria:

1. RG = HTP = HTB ≠ LTB; here RG is similar to HTP. Indel should be selected if call is similar in RG, HTP and HTB and alternative call in LTB.
2. HTP = HTB ≠ LTB = RG; here RG is similar to LTP. Indel should be selected if similar call is present in HTP and HTB and contrasting call in LTB and RG.

Further selected Indels based on above principles are subjected to chi‐square (χ^2^) analysis to check their goodness of fit ratio, that is 1:1 in HTB and LTB. A significant deviation from the normally expected ratio of any Indel would indicate the possible association with the target trait. Effect of significantly associated Indels on genes and genomic regions can be predicted through SnpEff (http://snpeff.sourceforge.net/) software.

### Application of Indel‐seq approach in pigeonpea

#### Extreme pools for Indel‐seq

**Figure 2:**
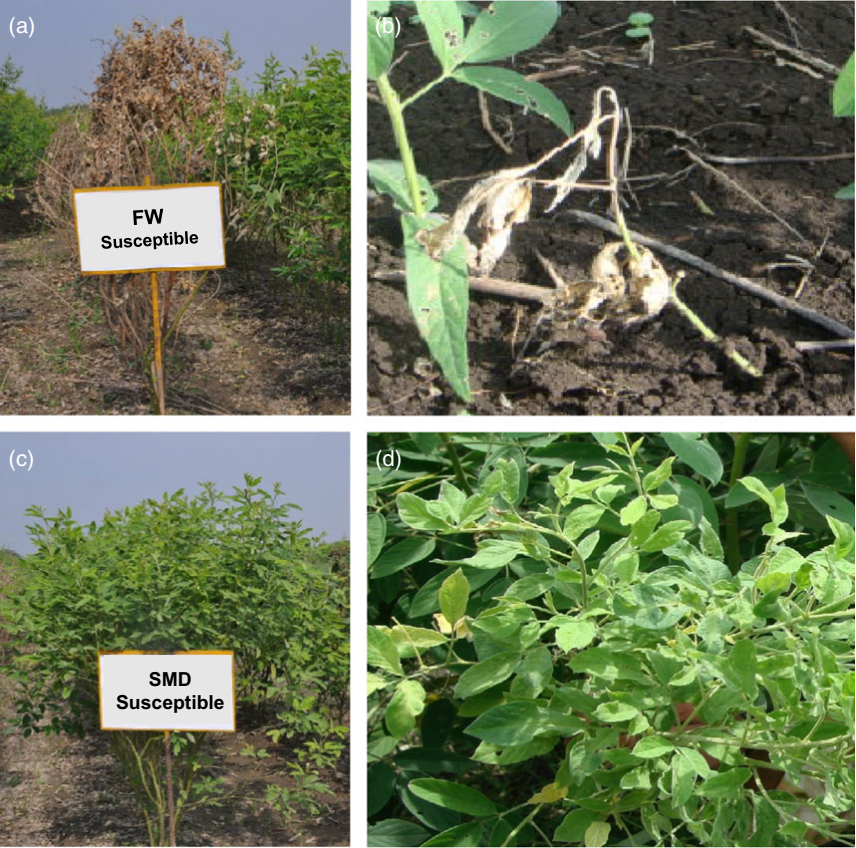
Phenotypic reaction of resistant and susceptible *Fusarium* wilt (FW) and sterility mosaic disease (SMD) plants. FW is a seed and soil borne fungal disease caused by *Fusairum udum*. Wilt symptoms usually appear when plants are in flowering and podding stage (a), but sometimes occur earlier when plants are 1‐2‐month‐old (b). SMD is a viral disease caused by *Pigeonpea sterility mosaic virus (PSMV)*. This disease can be easily identified from a distance as patches of bushy, pale green plants (c) without flower or pods (d). Due to excess vegetative growth, without growing into reproductive phase, this disease is known as the *green plague* of pigeonpea.

To deploy Indel‐seq in pigeonpea for detecting the candidate genomic regions/genes for FW and SMD resistance, available sequencing and phenotypic data were utilized in this study (Singh *et al*., 2016a). In brief, phenotyping data generated for resistance to FW and SMD on the RIL population, that is ICPL 20096 (resistant to FW and SMD, HTP) × ICPL 332 (susceptible to FW and SMD, LTP), were used for defining resistant bulk (HTB) and susceptible bulk (LTB) of 16 individual RILs in each group (Figures 2 and S1–S2). Using WGRS, a total of 9.27, 8.99 and 8.43 Gb data were generated for the resistant parent or HTP and HTB and LTB, respectively (Table S1). Cleaned data were aligned to the pigeonpea reference genome resulting in mapping of total 90.6% (HTP), 81.8% (HTB) and 82.5% (LTB) of the total high‐quality reads. Genome coverage was found to be 89.21% in HTP, 87.72% in HTB and 87.37% in LTB with an average depth of 13.4 X in HTP, 11.4 X in HTB and 10.8 X in LTB (Table S1).

#### Candidate Indels

Resequencing data sets for HTP, HTB and LTB were aligned with reference genome (RG) for identification of Indels (Varshney *et al*., 2012a). As a result, 211 603 genomewide Indels were identified. Of 211 603 Indels, 89 261 were identified on the pseudomolecules and the remaining were present on CcLG0 or floating scaffolds.

**Figure 3:**
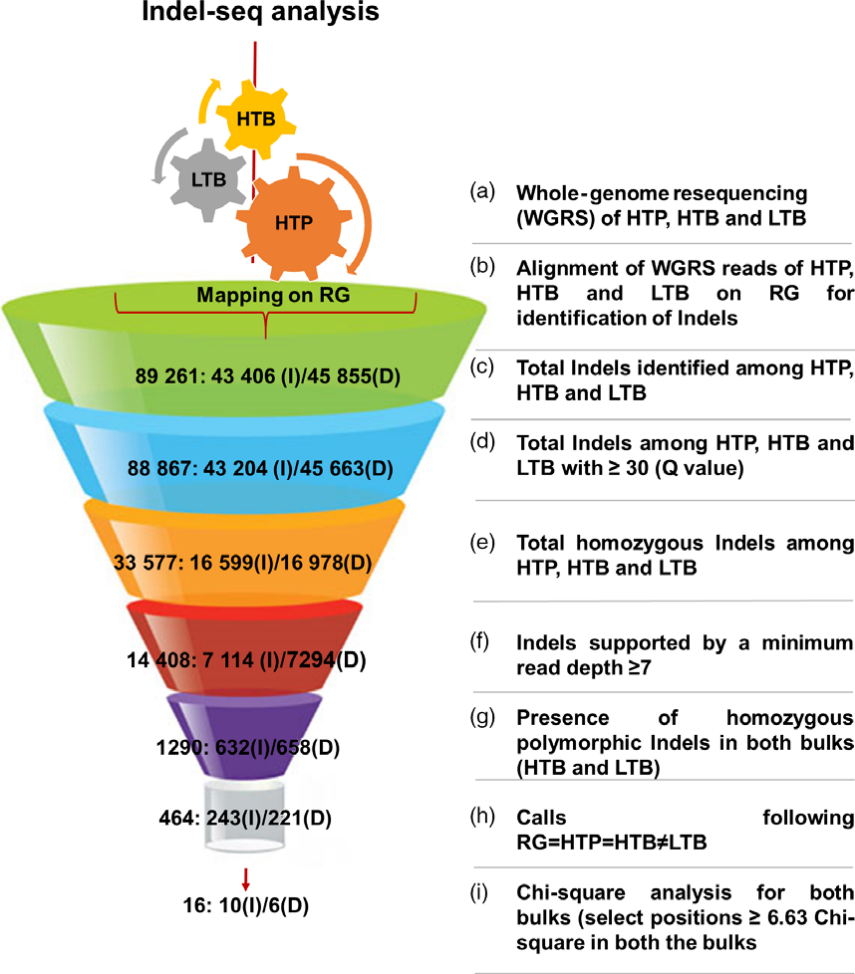
Flow diagram of Indel‐seq analysis for identification of candidate genes for FW and SMD resistance in pigeonpea. (a) Whole‐genome resequencing of the resistant parent (HTP), resistant bulk (HTB) and susceptible bulk (LTB) was performed with more than ≥10× genome coverage. (b) The generated raw reads of HTP, HTB and LTB were aligned with the reference genome (RG) for identification of genomewide Indels. The value presented in the funnel is the number of Indels identified/selected in each step, which is further classified as insertion (I) and deletion (D) (c) Total number of Indels identified after mapping of HTP, HTB and LTB on RG. (d) Further, only those Indels were selected, which possess ≥30 quality score. (e) Only homozygous Indels among HTP, HTB and LTB bulks were selected for further analysis (f) To remove false‐positive associations, only those Indels were selected which possesses reads ≥7 at both the bulk positions. (g) Homozygous polymorphic Indels were identified between both the bulks. (h) The classical concept of bulked segregant analysis (BSA) approach was implemented (RG = HTP = HTB ≠ LTB) for identification of putatively associated Indels (see text for the explanation). (i) Chi‐square test at 99% probability level was performed at each selected positions based on the presence of reads at selected Indel positions to select trait‐associated Indels.

A total of 88,867 Indels with Q value >30 were selected for downstream analysis (Table S2 and Figure S3‐S5). The lengths of these Indels were ranged from 1 bp to 99 bp (Figure S6). Indels with heterozygous and ‘N’ (missing) calls in the HTP, HTB and LTB were also discarded, and a set of 33 577 Indels was subjected to further filtration. After applying final filtering criteria, that is Indels with read depth ≥7 were selected, the number of Indels reduced to 14 408 across HTP, HTB and LTB. On pairwise analysis, a total of 1290 Indels were identified between HTB and LTB. These Indels were checked for the concept, that is RG = HTP = HTB ≠ LTB. As a result, 464 putative Indels were identified. Based on chi‐square test of the 464 Indels, only 16 Indels showed chi‐square values ≥6.63 depicted to have an association with traits of interest (Figure 3). The chi‐square values in HTB ranged from 7 (*P*‐value: 0.008151) to 12 (*P*‐value: 0.000532) and in LTB ranged from 7 (*P* value: 0.008151) to 14.22 (*P*‐value: 0.000163) (Table 1). These 16 Indels were found affecting 26 genes (Table 1). Of 26 affected genes, 24 genes showed the effect in the upstream/downstream of the genic region and two genes have effect at genic level (Table S3). Few of these candidate genes have been reported to play significant role in the defence mechanisms in other plant species (Table S3).

**Table 1:** Identification of Indels between resistant and susceptible bulks using Indel‐seq approach

Gene^a^	Type^b^	Linkage group	Position (bp)	RG^c^ base	HTP^d^ base	Resistant bulk				Susceptible bulk			
						HTB^e^ base	Read depth	χ^2^ value	*P* value	LTB^f^ base	Read depth	χ^2^ value	*P* value
*C.cajan_05665* (d)	I	CcLG02	12 535 647	C	C	C	15	8.07	<0.001	CA	14	9.94	<0.001
*C.cajan_05666* (u)													
*C.cajan_05815* (d)	D	CcLG02	14 020 849	CA	CA	CA	7	7.00	<0.001	C	15	8.00	<0.001
*C.cajan_05816* (u)													
*C.cajan_05857* (u)	I	CcLG02	14 397 213	A	A	A	19	8.89	<0.001	AT	18	7.12	<0.001
*C.cajan_05858* (d)													
*C.cajan_06311* (d)	I	CcLG02	19 386 341	T	T	T	14	7.14	<0.001	TC	11	8.00	<0.001
*C.cajan_09080* (u)	D	CcLG03	10 887 279	GTA	GTA	GTA	13	9.31	<0.001	G	16	9.80	<0.001
*C.cajan_11099* (d)	I	CcLG06	890 690	A	A	A	12	12.00	<0.001	AT	12	9.00	<0.001
*C.cajan_11101* (u)													
*C.cajan_11323* (u)	I	CcLG06	3 364 388	C	C	C	8	8.00	<0.001	CT	7	8.33	<0.001
*C.cajan_11324* (d)													
*C.cajan_17341* (i)	D	CcLG07	405 527	AT	AT	AT	8	8.00	<0.001	A	14	7.14	<0.001
*C.cajan_16014* (u)	I	CcLG08	7 106 619	T	T	T	8	8.00	<0.001	TG	20	7.00	<0.001
*C.cajan_16015* (d)													
C.cajan_16060 (d)	I	CcLG08	7 820 397	C	C	C	19	11.84	<0.001	CCAACAA	11	10.29	<0.001
*C.cajan_22308* (u)	I	CcLG09	2 209 342	A	A	A	14	7.14	<0.001	AT	11	11.00	<0.001
*C.cajan_22309* (d)													
*C.cajan_14502* (u)	I	CcLG10	13 435 965	C	C	C	13	9.31	<0.001	CA	17	8.07	<0.001
*C.cajan_14503* (d)													
*C.cajan_14515* (u)	D	CcLG10	13 516 086	TTA	TTA	TTA	15	8.07	<0.001	T	8	14.22	<0.001
*C.cajan_14516* (d)													
*C.cajan_15032* (f)	D	CcLG10	18 889 276	AT	AT	AT	18	8.00	<0.001	A	17	7.36	<0.001
*C.cajan_01566* (u)	D	CcLG11	17 030 340	CA	CA	CA	19	8.89	<0.001	C	8	7.36	<0.001
*C.cajan_01567* (d)													
*C.cajan_02069* (u)	I	CcLG11	22 814 098	G	G	G	15	8.07	<0.001	GT	11	7.36	<0.001

a Gene: u: upstream region; d: downstream region: i, intron; f, frame shift.

b Type of Indels: ‘I’ stand for insertion and ‘D’ stand for deletion.

c RG: Reference genome (Asha; ICPL 87119) (http://www.icrisat.org/gt-bt/iipg/genomedata.zip).

d HTP: Resistant parent (ICPL 20096).

e HTB: Resistant bulk.

f LTB: Susceptible bulk.

### Validation of candidate Indels

To validate and classify the identified 16 candidate Indels associated with the target genes for FW and SMD resistance, a comparative analysis based on allele frequencies in available sequence data was performed among four additional FW/SMD‐resistant and FW/SMD‐susceptible genotypes along with HTP, RG, HTB and LTB (Table 2). As a result, of 16 candidate Indels, five with an effect on eight candidate genes were validated (Table 2).

**Table 2:** Validation of candidate Indels in four known (resistant and susceptible) genotypes for FW and SMD resistance

Linkage group	Indel positions (bp)	RG^a^		HTP^b^		HTB^c^		LTB^d^		ICPB 2049		ICPL 99050		ICPL 20097		ICP 8863		*P*‐value for FW resistance	*P*‐value for SMD resistance
		FW‐R^e^	SMD‐R^e^	FW‐R^e^	SMD‐R^e^	FW‐R^e^	SMD‐R^e^	FW‐S^f^	SMD‐S^f^	FW‐S^f^	SMD‐R^e^	FW‐R^e^	SMD‐R^e^	FW‐R^e^	SMD‐R^e^	FW‐R^e^	SMD‐S^f^		
CcLG02	12 535 647	C		C		C		CA		CA		C^g^		C		C		**<0.00**	0.42
CcLG02	14 020 849	CA		CA		CA		C		CA		CA		CA		C		0.42	**<0.00**
CcLG02	14 397 213	A		A		A		AT		A		A		A		A		0.08	0.08
CcLG02	19 386 341	T		T		T		TC		T		T		T		T		0.08	0.08
CcLG03	10 887 279	GTA		GTA		GTA		G		G		G		G		G		0.27	0.27
CcLG06	890 690	A		A		A		AT		A		A		A		A		0.08	0.08
CcLG06	3 364 388	C		C		C		CT		C		C		C		C		0.08	0.08
CcLG07	405 527	AT		AT		AT		A		A		AT		AT		AT		**<0.00**	0.42
CcLG08	7 106 619	T		T		T		TG		TG		T		T		T		**<0.00**	0.42
CcLG08	7 820 397	C		C		C		CCAACAA		C		C		C		C		0.08	0.08
CcLG09	2 209 342	A		A		A		AT		AT		AT		AT		AT		0.27	0.27
CcLG10	13 435 965	C		C		C		CA		C		C		C		C		0.08	0.08
CcLG10	13 516 086	TTA		TTA		TTA		T		TTA		TTA		TTA		TTA		0.08	0.08
CcLG10	18 889 276	AT		AT		AT		A		AT		AT^g^		AT		A^g^		0.04	**<0.00**
CcLG11	17 030 340	CA		CA		CA		C		C		C		C		C		0.27	0.27
CcLG11	22 814 098	G		G		G		GT		G		G		G		G		0.08	0.08

a RG: Reference genome (Asha; ICPL 87119) (http://www.icrisat.org/gt-bt/iipg/genomedata.zip).

b HTP: Resistant parent (ICPL 20096).

c HTB: Resistant bulk.

d LTB: Susceptible bulk.

e R: resistant reaction.

f S: susceptible reaction.

g Heterozygous calls.

*P*‐value <0.00 (boldface) found significant for specific disease resistance.

#### Indels for FW resistance

Three Indels, one each on CcLG02, CcLG07 and CcLG08, were found to be associated with FW resistance. For instance, one‐bp deletion identified on CcLG02 (at position 1 253 647 bp) showed ‘C’ allele in FW‐resistant genotypes and HTB, whereas ‘CA’ allele was present in LTB and FW‐susceptible genotype (ICPB 2049) with a *P*‐value <0.001. The identified one‐bp insertion in susceptible genotypes (‘C’ to ‘CA’) was found to be affecting AP‐1 complex subunit sigma‐2 (*C.cajan_05665*) and L‐ascorbate oxidase (*C.cajan_05665*) at upstream and downstream regions, respectively. At 405 527bp position on CcLG07, ‘AT’ allele was identified in HTB‐ and FW‐resistant genotypes and ‘A’ allele identified in LTB and susceptible genotype (ICPB 2049) with a *P*‐value of <0.001. This single‐bp deletion (‘AT’ to ‘A’) in susceptible genotypes showed an effect at intronic region and targeting receptor‐like protein kinase (*C.cajan_17341*). On CcLG08 (at position 7 106 619 bp), one‐bp deletion was observed in HTB‐ and FW‐resistant genotypes (‘T’ allele) in comparison with LTB and FW‐susceptible genotypes (‘TG’ allele) (with *P*‐value <0.001). The insertion of one bp (‘T’ to ‘TG’) in susceptible genotypes has shown the effect on two genes (*C.cajan_16014*; Transcriptional corepressor SEUSS and *C.cajan_16015*; Uncharacterized protein).

#### Indels for SMD resistance

For SMD resistance, Indel‐seq analysis has provided two associated Indels, one each on CcLG02 and CcLG10. On CcLG02 at 14 020 849 bp position, one‐bp insertion in HTB and SMD resistance genotypes (‘CA’ allele) was detected. In the case of LTB and susceptible genotype (ICP 8863), ‘C’ allele was present (with *P*‐value <0.001). The identified one‐bp deletion (‘CA’ to ‘C’) in susceptible genotypes targeting two genes (*C.cajan_05815* at upstream and *C.cajan_05816* at downstream region), and both the genes were annotated as conserved oligomeric Golgi complex subunit 5. Similarly, On CcLG10 (at position 18 889 276 bp) one‐bp insertion was observed in HTB‐ and SMD‐resistant genotypes (‘AT’ allele) in comparison with LTB and SMD‐susceptible genotype (‘A’ allele) (with *P*‐value <0.001). This single‐bp deletion in susceptible genotypes (‘AT’ to ‘A’) showed frame‐shift effect in an uncharacterized protein (*C.cajan_15032*).

## Discussion

NGS‐based genome mapping enables identification of candidate genomic regions/genes in a rapid way, which is often difficult using traditional methods in terms of time and resources required (Varshney *et al*., 2012b). Recently, a number of SNP‐based approaches combining BSA and WGRS have been successfully developed and implemented to identify the target candidate genes (see Zou *et al*. 2016). In the present study, an Indel‐seq approach has been proposed for the identification of candidate genes/Indels associated with target traits. This approach has been tested in pigeonpea for rapid identification of candidate genes associated with the FW and SMD resistance.

To enable WGRS‐based identification of candidate genes using mapping‐by‐sequencing approach, several methods have been developed and discussed in different crops (Abe *et al*., 2012; Austin *et al*., 2011; Hartwig *et al*., 2012; Nordström *et al*., 2013; Schneeberger *et al*., 2009; Takagi *et al*., 2013a; Trick *et al*., 2012). Based on the published literature and through large‐scale simulation studies, James *et al*. (2013) developed user guide for mapping‐by‐sequencing. Among different NGS‐based approaches, QTL‐seq approach provided the first successful example of mapping candidate genomic regions through NGS‐based approach in crop species like rice (Takagi *et al*., 2013b). QTL‐seq approach was found successful for identification of candidate genomic regions (SNPs) for FW and SMD resistance in pigeonpea (Singh *et al*., 2016a) and 100‐seed weight and root trait ratio (RTR %) in chickpea (Singh *et al*., 2016b). However, in the majority of above‐mentioned studies, Indels have been ignored. For effective applications of Indels in trait mapping, we propose here Indel‐seq approach that is a combination of WGRS and BSA. Deployment of Indel‐seq approach has been used for identification of candidate genomic regions associated with FW and SMD resistance in the present study.

### Application of Indel‐seq approach for identification of trait‐associated Indels

Two types of genetic variations, namely SNPs and Indels, are the most promising variations and used in the trait mapping studies in a number of crops (Huang *et al*., 2012; Li *et al*., 2013; Thudi *et al*., 2014). In the recent past, NGS‐based trait mapping approaches utilizing a large number of SNPs generated through resequencing/genotyping have been used for trait mapping (Varshney *et al*., 2014). SNP‐based mapping approaches identified candidate genes for the target traits in many reports but identification of a large number of cloned genes with the presence of functional Indels through map‐based cloning experiments for different traits in different crops revealed the importance of Indels for trait mapping and development of functional markers (Kage *et al*., 2015).

Comparative analysis of Indel‐seq approach with other NGS‐based QTL mapping approaches combining WGRS and BSA revealed some pros and cons over other methods of trait mapping (Table S4). The additional advantage of Indel‐seq mapping approach is to map the candidate genes in the population developed by crossing gamma‐induced mutants with the wild types due to the presence of genomewide Indels in the genome. Another important feature of Indel‐seq is the high probability of development of PCR‐based markers for trait mapping. The rapid fall in the cost of sequencing will facilitate application of Indel‐seq for trait mapping in diploid crops with relatively smaller genomes such as rice (389 Mb), chickpea (738 Mb), sorghum (818 Mb), pigeonpea (833 Mb). However, analysis of data sets for complex and large genome species requires some additional modification in the selection criteria of Indel for marker–trait association analysis.

Indel‐seq analysis in pigeonpea for mapping FW and SMD resistance has been very effective as it overcomes many constraints like identification of polymorphic markers between parents, the time required for genotyping of the mapping population, preparation of the (low density) genetic maps, and identification of QTLs (with large intervals). WGRS data of parental line and bulks revealed a higher number of genomewide Indels; however, comparatively low mapping percentage and genome coverage was obtained after aligning the raw sequences to the reference genome. This lower mapping and coverage percentage might be due to sequencing library used, sequencing errors, structural rearrangements or insertions in the query genome or deletions in the reference, a high percentage of repetitive elements (Sims *et al*., 2014) and quality of the reference genome. WGRS analysis of resistant parent and both the bulks revealed 89 261 genomewide Indels and 33 577 Indels between the bulks (HTB vs LTB), which further narrowed down to 1290 Indels, based on stringent selection criteria (read depth and homozygosity of calls in the bulks). The number of Indels was further reduced to 464 based on Indel‐seq principle. However, this number is comparatively higher than the previous SMD resistance mapping experiments in which only 120 and 78 SSRs were found polymorphic in two mapping populations after screening of 3000 SSR markers (Gnanesh *et al*., 2011). Finally, based on chi‐square analysis 16 candidate Indels were identified targeting 26 different candidate genes. The Indel‐seq pipeline discussed in this report is very simple and after mapping raw reads to the reference genome, analysis can be done using simple Perl Script or in Microsoft Excel program (2010 and above).

### Identification of significant genomic regions for FW‐ and SMD‐resistant breeding

To check the efficiency of Indel‐seq in identifying possible candidates (markers/genes) for the target traits, we have also used identified Indels (Table S5) in a recently proposed method known as EXPLoRA‐web BSA (Duitama *et al*., 2014). We have significant results from each of the models proposed in EXPLoRA‐web BSA (Tables S6). As expected, the lowest number of QTLs was reported in the high sensitive model (α = 5, β = 1) and highest number of QTLs in the high specific model (α = 30, β = 1). Interestingly, 12 of 16 candidate Indels identified through Indel‐seq approach were found common in EXPLoRA‐web BSA analysis (Table S7). Moreover, from the five validated Indels in the present study, four were also found in EXPLoRA‐web BSA analysis. This has enhanced our confidence in proposing Indel‐seq as a possible approach for fast trait mapping experiments. However, it is important to mention that EXPLoRA‐web BSA has provided a large number of possible Indels’ associations, which directly cannot be applied for genomics‐assisted breeding (GAB) programmes, whereas Indel‐seq has provided reasonable numbers of high confidence MTAs (three for FW and two for SMD) which can be converted into KASP markers. After validation of KASP markers, it can be utilized in GAB for development of FW‐ and SMD‐resistant pigeonpea genotypes.

## Conclusions

It is evident from the present study that identification of candidate genes for targeted traits based on NGS will not only increase the precision and power but also generate results in less time than the conventional methods of genome mapping. In near future due to rapid declining of sequencing cost and availability of high‐quality draft genome sequences in several crops, we envisage application of Indel‐seq for trait mapping and GAB for crop improvement. Identified target genes and associated Indels in the present study were validated on defined sets of genotypes for which sequence data were available. These results after validation on larger sets of genotypes will be useful in guiding diseases resistance breeding efforts in pigeonpea.

## Materials and methods

### Plant materials and construction of pools

Six pigeonpea genotypes were selected based on their FW and SMD responses identified from our previous experiments (Saxena *et al*., 2010a; Singh *et al*., 2016a; Varshney *et al*., 2012a). Among the selected genotypes, ICPL 20096, ICPL 20097, ICPL 8863, ICPL 99050 and ICPL 87119 were FW resistant and ICPL 20096, ICPL 20097, ICPL 99050, ICPB 2049 and ICPL 87119 were SMD resistant. Similarly, among the six genotypes, ICPB 2049 was FW susceptible, and ICP 8863 was SMD susceptible. Two genotypes ICPL 20096 (FW and SMD resistant) and ICP 332 (FW and SMD susceptible) with contrasting phenotypes were crossed and selfed through single seed descent method to develop 188 F_7_ recombinant inbred lines (RILs).

These RILs were phenotyped for FW and SMD resistance using standard procedures as mentioned in Nene and Reddy (1976) and Singh *et al*. (2003). The detailed descriptions on sick plot nursery, filed design and construction of bulks have been presented in Singh *et al*. (2016a).

### Sequencing libraries and alignment of short reads of bulks

Raw sequencing data of ICPL 20096 (resistant parent or HTP) and resistant bulk or HTB and susceptible bulk or LTB were used for Indel‐seq analysis (Table S2). The generated paired end reads of 251 bp lengths were cleaned using the tool Sickle (Joshi and Fass, 2011) with minimum phred quality score of 30 and minimum read length of 70 bp. The reads containing ‘Ns’ were also removed. The clean data of samples were used to align to the pigeonpea reference genome (Varshney *et al*., 2012a) using BWA: Burrows–Wheeler Aligner (Li and Durbin, 2009) to get the Sequence Alignment/Map (SAM)/BAM (Binary Alignment/Map) alignment files, which results in alignment files in BAM format. The BAM files were further processed for Indel realignment using IndelRealigner component of Genome Analysis Toolkit (GATK; McKenna *et al*., 2010), and Picard utility was used for adding read group information. These processed BAM files were then subjected for the variants calling through GATK (DePristo *et al*., 2011) using standard parameters for the parent and both the bulks. The identified genomewide variants were further used for Indel‐seq analysis for the identification of MTAs.

### Mining of resequencing data sets for validation

To validate the candidate SNPs, resequencing data sets of four genotypes, namely ICPL 20097 (R‐FW and R‐SMD) and ICP 8863 (R‐FW and S‐SMD), ICPB 2049 (S‐FW and R‐SMD) and ICPL 99050 (R‐FW and R‐SMD), were used to find out the genes/markers unique to FW and SMD (Kumar *et al*., 2016). To test the association, *p‐*value was calculated between identified Indels with the target traits using single factor ANOVA in Microsoft Excel 2013.

### EXPLoRA‐web BSA

EXPLoRA‐web BSA works upon the principle of LD to detect QTLs using Hidden Markov Model (HMM) (Duitama *et al*., 2014). Genomewide mapping reads of susceptible bulk (LTB) onto the reference genome (RG) was utilized to develop input files for EXPLoRA‐web BSA analysis. Only those positions were selected for analyses, which were supported by a minimum of 10 reads. LTB was chosen for BSA in the present analysis because RG was resistant to both the diseases (FW and SMD). To control the EXPLoRA‐web models, three different parameters were utilized for identification of QTLs (i) α = 5; β = 1 (high sensitivity) (ii) α = 10; β = 1 (the middle ground between sensitivity and specificity) and (iii) α=30; β=1 (high specificity). The α/β ratio determines the shape of the β distribution in the models, which reflects the probability for the phenotype‐linked states (Pulido‐Tamayo *et al*., 2016).

## Conflict of interest

The author(s) declares that they have no competing interests.

## Supporting information


**Figure S1** Classification of RILs and parents based on FW percent disease score (PDI).
**Figure S2** Classification of RILs and parents based on SMD percent disease score (PDI).
**Figure S3** Genome‐wide identified insertion plots for all linkage groups.
**Figure S4** Genome‐wide identified deletion plots for all linkage groups.
**Figure S5** Genome‐wide identified Indels plots for all linkage groups.
**Figure S6** Number and length of insertions and deletions identified after mapping.
**Table S1** Summary of Illumina sequencing and mapping of parental lines and bulks.
**Table S2** Linkage group wise distribution of genome wide Indels.
**Table S3** Annotation of identified putative candidate genes associated with FW and SMD resistance.
**Table S4** Mapping information of susceptible bulk (HTB) onto the reference genome (RG).
**Table S5** List of putative associated QTLs identified through EXPLoRA‐web BSA analysis.
**Table S7** Comparison of BSA‐based WGRS approaches for trait mapping.

**Table S6** Comparison of the identified genomic regions from Indel‐seq with other mapping approaches.
