# Supplementary material for: Indel‐seq: a fast‐forward genetics approach for identification of trait‐associated putative candidate genomic regions and its application in pigeonpea (Cajanus cajan)
Source: Plant Biotechnol J. 2017 Feb 9;15(7):906–14. doi: 10.1111/pbi.12685 (PMC5466435; doi:10.1111/pbi.12685)
Supplement: Supplementary file 1 — Figure S1 Classification of RILs and parents based on FW percent disease score (PDI). Figure S2 Classification of RILs and parents based on SMD percent disease score (PDI). Figure S3 Genome‐wide identified insertion plots for all linkage groups. Figure S4 Genome‐wide identified deletion plots for all linkage groups. Figure S5 Genome‐wide identified Indels plots for all linkage groups. Figure S6 Number and length of insertions and deletions identified after mapping. Table S1 Summary of Illumina sequencing and mapping of parental lines and bulks. Table S2 Linkage group wise distribution of genome wide Indels. Table S3 Annotation of identified putative candidate genes associated with FW and SMD resistance. Table S4 Mapping information of susceptible bulk (HTB) onto the reference genome (RG). Table S5 List of putative associated QTLs identified through EXPLoRA‐web BSA analysis. Table S7 Comparison of BSA‐based WGRS approaches for trait mapping. [file PBI-15-906-s001.docx]

**Supplementary Information**

Indel-seq: a fast forward genetics approach for identification of trait associated putative candidate genomic regions and its application in pigeonpea (*Cajanus cajan*)

Vikas K Singh^1, †^, Aamir W Khan^1, †^, Rachit K Saxena^1,†^, Pallavi Sinha^1,†^, Sandip M Kale^1^, Swathi Parupalli^1^, Vinay Kumar^1^, Annapurna Chitikineni^1^, V Suryanarayana^1^, C V Sameer Kumar^1^, Mamta Sharma^1^, G Anuradha^2^, K N Yamini^2^, S Munniswamy^3^, Rajeev K Varshney^1,4,*^

^1^International Crops Research Institute for the Semi-Arid Tropics, Patancheru- 502324, Telangana State, India

^2^Agricultural Research Station (ARS)-Tandur, Professor Jayashankar Telangana State Agricultural University (PJTSAU),Hyderabad- 501141, Telangana State, India

^3^Agricultural Research Station (ARS)-Gulbarga, University of Agricultural Sciences (UAS), Raichur- 585101, Karnataka, India

^4^School of Plant Biology and Institute of Agriculture, The University of Western Australia, Crawley- 6009, WA, Australia

†Authors contributed equally to this work

**Short Summary:** This study proposes a new trait mapping approach termed as Indel-seq, which relies on whole genome re-sequencing, bulked segregant analysis and identification of Indels. This approach has been successfully applied in pigeonpea (*Cajanus cajan*) for the identification of putative candidate genes for resistance to fusarium wilt and sterility mosaic disease.

*Author for Correspondence

Rajeev K Varshney

International Crops Research Institute for the Semi-Arid Tropics (ICRISAT)

Patancheru - 502 324, India

Telephone: 91-40-30713305;

Fax: 91-40-30713074

Email: [r.k.varshney@cgiar.org](mailto:r.k.varshney@cgiar.org)

**Table S1** Summary of Illumina sequencing and mapping reads of parental line and bulks on reference genome

| Genotype | Data generated (Gb) | Alignment percent to the RG^a^ | Percent coverage of genome | Mean depth (X) |
| --- | --- | --- | --- | --- |
| Resistant parent (HTP)^b^ | 9.27 | 90.6 | 89.21 | 13.4 |
| Resistant bulk (HTB)^c^ | 8.99 | 81.8 | 87.72 | 11.4 |
| Susceptible bulk (LTB)^d^ | 8.43 | 82.5 | 87.37 | 10.8 |

^a^Short reads of HTP, HTB, and LTB, were aligned to the publicly available pigeonpea reference genome (RG) of Asha (ICPL 87119; Varshney et al. 2012)

(<http://www.icrisat.org/gt-bt/iipg/genomedata.zip>).

^b^Resistant parent for FW and SMD resistance (ICPL 20096) of mapping population

^c^HTB was constituted using 16 resistant RILs identified at both locations (Patancheru and Gulbarga)

^d^LTB was constituted using 16 susceptible RILs identified at both locations (Patancheru and Gulbarga)

**Table S2** Linkage group wise distribution of genome wide Indels

| Linkage  group | Indels | Insertions | Deletion | Indels/Kb |
| --- | --- | --- | --- | --- |
| CcLG01 | 7417 | 3639 | 3778 | 0.42 |
| CcLG02 | 12492 | 6023 | 6469 | 0.34 |
| CcLG03 | 9935 | 4856 | 5079 | 0.34 |
| CcLG04 | 5265 | 2601 | 2664 | 0.42 |
| CcLG05 | 2133 | 1054 | 1079 | 0.42 |
| CcLG06 | 9401 | 4600 | 4801 | 0.40 |
| CcLG07 | 7162 | 3392 | 3770 | 0.37 |
| CcLG08 | 7233 | 3555 | 3678 | 0.35 |
| CcLG09 | 3316 | 1591 | 1725 | 0.32 |
| CcLG10 | 7997 | 3848 | 4149 | 0.35 |
| CcLG11 | 16516 | 8045 | 8471 | 0.34 |
| Average | 8078.82 | 3927.64 | 4151.18 | 0.37 |
| Total | 88867 | 43204 | 45663 | 0.36 |

**Table S3** Annotation of identified putative candidate genes associated with resistance to FW and SMD

| Linkage  group | Position (bp) | Gene id | Effect | Uniprot id | Protein name |
| --- | --- | --- | --- | --- | --- |
| CcLG02 | 12535647 | *C.cajan_05665* | Downstream | O23685 | AP-1 complex subunit sigma-2 (Adaptor AP-1 19 kDa protein) |
|  |  | *C.cajan_05666* | Upstream | Q40588 | L-ascorbate oxidase (ASO) (Ascorbase) |
| CcLG02 | 14020849 | *C.cajan_05815* | Downstream | Q8C0L8 | Conserved oligomeric Golgi complex subunit 5 |
|  |  | *C.cajan_05816* | Upstream | Q9UP83 | Conserved oligomeric Golgi complex subunit 5 |
| CcLG02 | 14397213 | *C.cajan_05857* | Upstream | P87027 | Septum-promoting GTP-binding protein 1 (GTPase spg1) (Sid3 protein) |
|  |  | *C.cajan_05858* | Downstream | P51910 | Apolipoprotein D (Apo-D) (ApoD) |
| CcLG02 | 19386341 | *C.cajan_06311* | Downstream | Q766C2 | Aspartic proteinase nepenthesin-2 |
| CcLG03 | 10887279 | *C.cajan_09080* | Upstream | P92934 | Amino acid permease 6 (Amino acid transporter AAP6) |
| CcLG06 | 890690 | *C.cajan_11099* | Downstream | I1K982 | Bromo-adjacent homology (BAH) domain-containing protein |
|  |  | *C.cajan_11101* | Upstream | I1JUR1 | Formin-like protein 8-like [Glycine max] |
| CcLG06 | 3364388 | *C.cajan_11323* | Upstream | P04323 | Retrovirus-related Pol polyprotein from transposon 17.6 |
|  |  | *C.cajan_11324* | Downstream | S1RW12 | Retrotransposon protein, putative, Ty3-gypsy subclass |
| CcLG07 | 405527 | *C.cajan_17341* | Intronic | Q9FID5 | Probable receptor-like protein kinase At5g39030 |
| CcLG08 | 7106619 | *C.cajan_16014* | Upstream | Q8W234 | Transcriptional corepressor SEUSS (AtSEU) |
|  |  | *C.cajan_16015* | Downstream | I1MQ19 | Uncharacterized protein |
| CcLG08 | 7820397 | *C.cajan_16060* | Downstream | G7JVP1 | DUF4283 domain protein |
| CcLG09 | 2209342 | *C.cajan_22308* | Upstream | Q9SZC9 | Copper-transporting ATPase PAA1 |
|  |  | *C.cajan_22309* | Downstream | Q6NPP4 | Calmodulin-binding transcription activator 2 |
| CcLG10 | 13435965 | *C.cajan_14502* | Upstream | P48528 | Serine/threonine-protein phosphatase PP-X isozyme 2 |
|  |  | *C.cajan_14503* | Downstream | M5VVF0 | Putative nuclease HARBI1-like [Glycine max] |
| CcLG10 | 13516086 | *C.cajan_14515* | Upstream | Q9C9H7 | Receptor-like protein 12 (AtRLP12) |
|  |  | *C.cajan_14516* | Downstream | Q9C9H7 | Receptor-like protein 12 (AtRLP12) |
| CcLG10 | 18889276 | *C.cajan_15032* | Frame-shift | M5VJF1 | Uncharacterized protein |
| CcLG11 | 17030340 | *C.cajan_01566* | Upstream | P49045 | Vacuolar-processing enzyme (VPE) |
|  |  | *C.cajan_01567* | Downstream | Q9FWX2 | NAC domain-containing protein 7 |
| CcLG11 | 22814098 | *C.cajan_02069* | Upstream | B9I3X5 | CASP-like protein 1F2 (PtCASPL1F2) |

**Table S4** Comparison of BSA-based WGRS approaches for trait mapping

| Description | Indel-seq | Seq-BSA | QTL-seq | MULTIPOOL | X-QTL |
| --- | --- | --- | --- | --- | --- |
| Application to isolation of gene with small effect | Yes | Yes | Yes | Yes | Yes |
| Mapping candidate gene(s) in EMS induced population | No | Yes | Yes | Yes | Yes |
| Mapping candidate gene(s) in fast neutron/ gamma irradiated population | Yes | No | No | No | No |
| Successful example (with genome size) | Pigeonpea  (833 Mb) | Pigeonpea  (833 Mb) | Rice  (394 Mb) | Yeast  (12.5 Mb) | Yeast  (12.5 Mb) |
| Variants used for mapping | Indels | SNPs | SNPs | SNPs | SNPs |
| Number of total variants used as marker | 89,261 | 35,877 | 161,563 | NA | NA (180X coverage) |
| Number of progenies used for bulking | 16 | 16 | 20 | 31 | 10^5†^ |
| Approach | RG=HTP=HTB≠LTB | HTP=HTB≠LTB and SNP index | SNP index | Allele frequency | SNP frequency |
| Reference | Present study | Singh et al. (2016) | Takagi et al. (2012) | Edwards and Gifford et al. (2012) | Ehrenreich et al. (2010) |

RG: reference genome; HTP: high trait parent; HTB: high trait bulk; LTB: low trait bulk; X-QTL: Extreme QTL; ^†^Can perform X-QTL mapping with up to 10^5^ individuals

**Table S5** Summary of mapping susceptible bulk (LTB) reads onto the reference genome (RG)

| Linkage  group | Total reads | Total high-quality reads (≥ 10 X coverage)^†^ | Percent high quality reads | Range of read depth (X coverage) |
| --- | --- | --- | --- | --- |
| CcLG01 | 6384 | 1639 | 25.67% | 10-227 |
| CcLG02 | 10605 | 2643 | 24.92% | 10-247 |
| CcLG03 | 8507 | 2405 | 28.27% | 10-212 |
| CcLG04 | 4657 | 1377 | 29.57% | 10-183 |
| CcLG05 | 1802 | 537 | 29.80% | 10-226 |
| CcLG06 | 8069 | 1965 | 24.35% | 10-64 |
| CcLG07 | 6094 | 1665 | 27.32% | 10-184 |
| CcLG08 | 6117 | 1978 | 32.34% | 10-210 |
| CcLG09 | 2964 | 692 | 23.35% | 10-64 |
| CcLG10 | 6426 | 1495 | 23.26% | 10-233 |
| CcLG11 | 13654 | 3486 | 25.53% | 10-248 |

^†^High-quality Indel reads were utilized for EXPLoRA-web BSA analysis

**Table S6** List of putative associated QTLs identified through EXPLoRA-web BSA analysis

**Detailed Excel file has been attached.**

**Table S7** Comparison of the identified genomic regions from Indel-seq with other mapping approaches

| Linkage group/  No of significant positions or QTLs identified | Indel-seq (Current study)  physical position (bp) | Seq-BSA^a^ | EXPLoRA-web BSA | | |
| --- | --- | --- | --- | --- | --- |
|  |  | Physical position (bp) | α=5 & β=1  Physical position (bp) | α=10 & β=1  Physical position (bp) | α=30 & β=1  Physical position (bp) |
|  | 16 | 8 | 9 | 34 | 211 |
| CcLG02 | 12535647 |  |  | ++ | ++ |
| CcLG02 | 14020849 |  | ++ | ++ | ++ |
| CcLG02 | 14397213 |  | ++ | ++ | ++ |
| CcLG02 | 19386341 |  |  |  | ++ |
| CcLG03 | 10887279 |  |  |  |  |
| CcLG06 | 890690 |  |  |  |  |
| CcLG06 | 3364388 |  |  |  | ++ |
| CcLG07 | 405527 |  |  |  |  |
| CcLG08 | 7106619 |  |  |  | ++ |
| CcLG08 | 7820397 |  |  |  |  |
| CcLG09 | 2209342 |  | ++ | ++ |  |
| CcLG10 | 13435965 |  |  | ++ | ++ |
| CcLG10 | 13516086 |  |  | ++ | ++ |
| CcLG10 | 18889276 |  | ++ |  | ++ |
| CcLG11 | 17030340 | ++ | ++ | ++ | ++ |
| CcLG11 | 22814098 | ++ |  | ++ | ++ |
| Overlapping regions | | 2 | 5 | 8 | 11 |

^a^ Singh et al. (2016)

++ indicates ~3.0 Mb flanking regions overlapped between Indel-seq positions with other mapping experiments


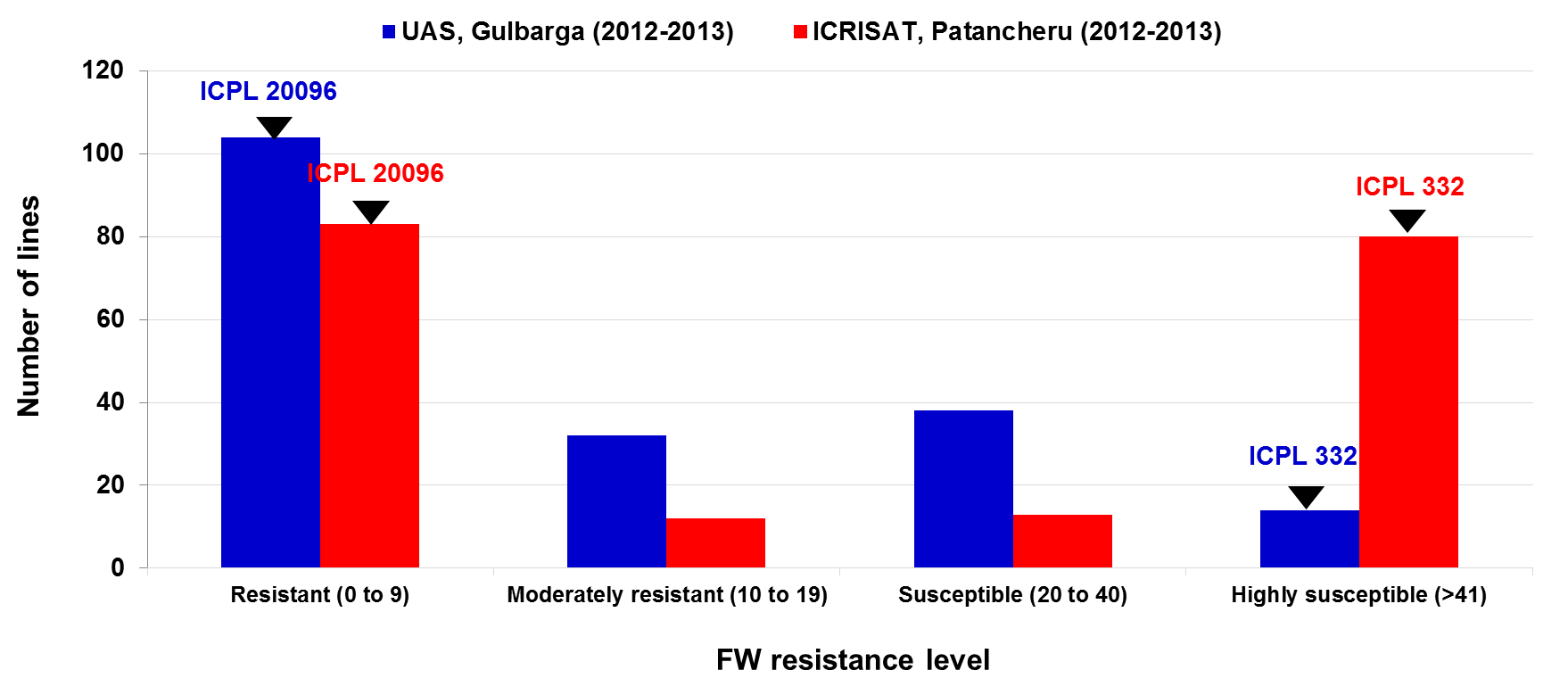


**Figure S1 Classification of RILs and parents based on FW percent disease score (PDI)**

Classification of 188 RILs derived from a cross between ICPL 20096 and ICPL 332 based on FW PDI score. This RILs population was phenotyped at multi-location environment at UAS, Gulbarga, and ICRISAT, Patancheru. X-axis represents the classification of lines based on FW score, and Y-axis represents a number of lines falling into each level. The arrow indicates the mean FW score of resistant and susceptible parents


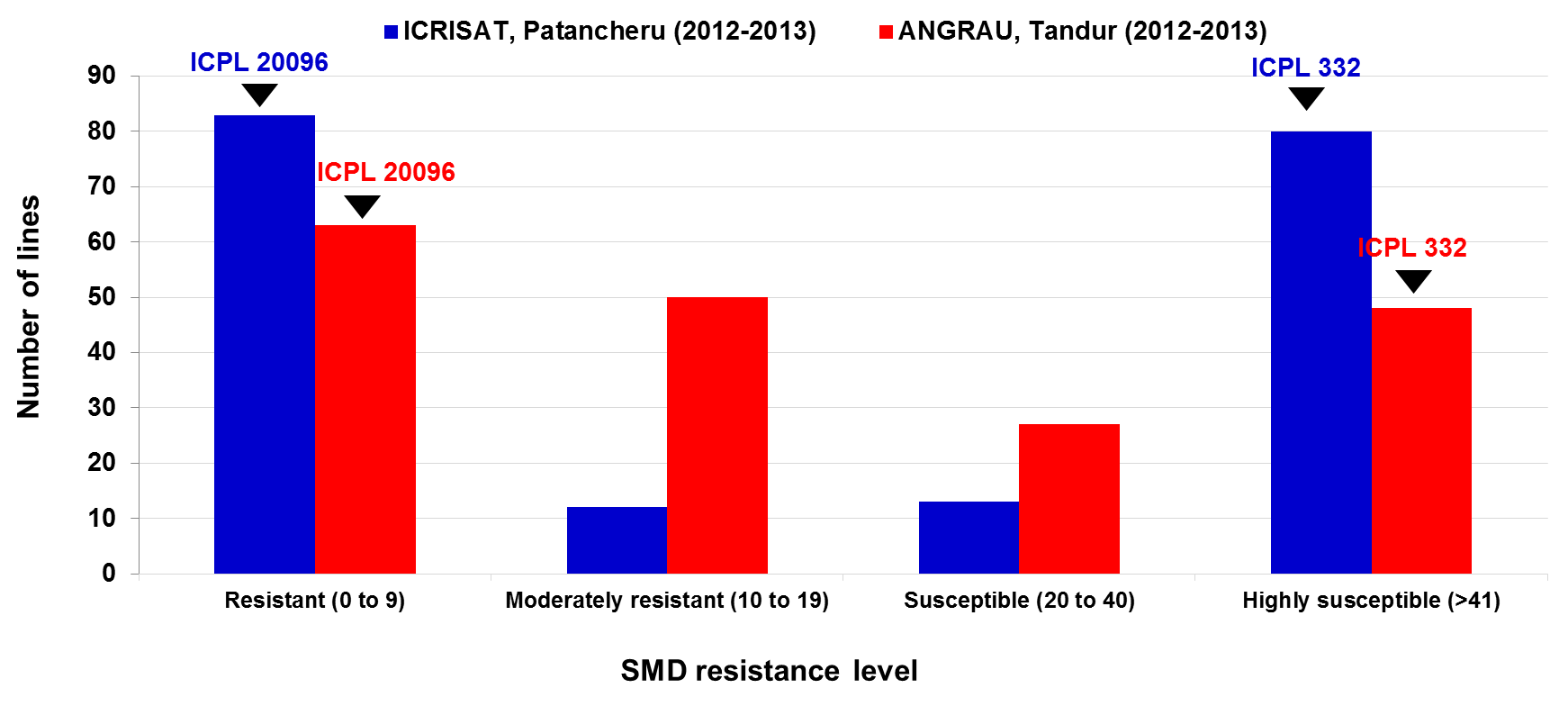


**Figure S2 Classification of RILs and parents based on SMD percent disease score (PDI)**

Classification of 188 RILs derived from a cross between ICPL 20096 and ICPL 332 based on SMD PDI score. This RILs population was phenotyped at multi-location environment at ICRISAT, Patancheru, and ANGRAU, Tandur. X-axis represents the classification of lines based on SMD score, and Y-axis represents a number of lines falling into each level. The arrow indicates the mean SMD score of resistant and susceptible parents.


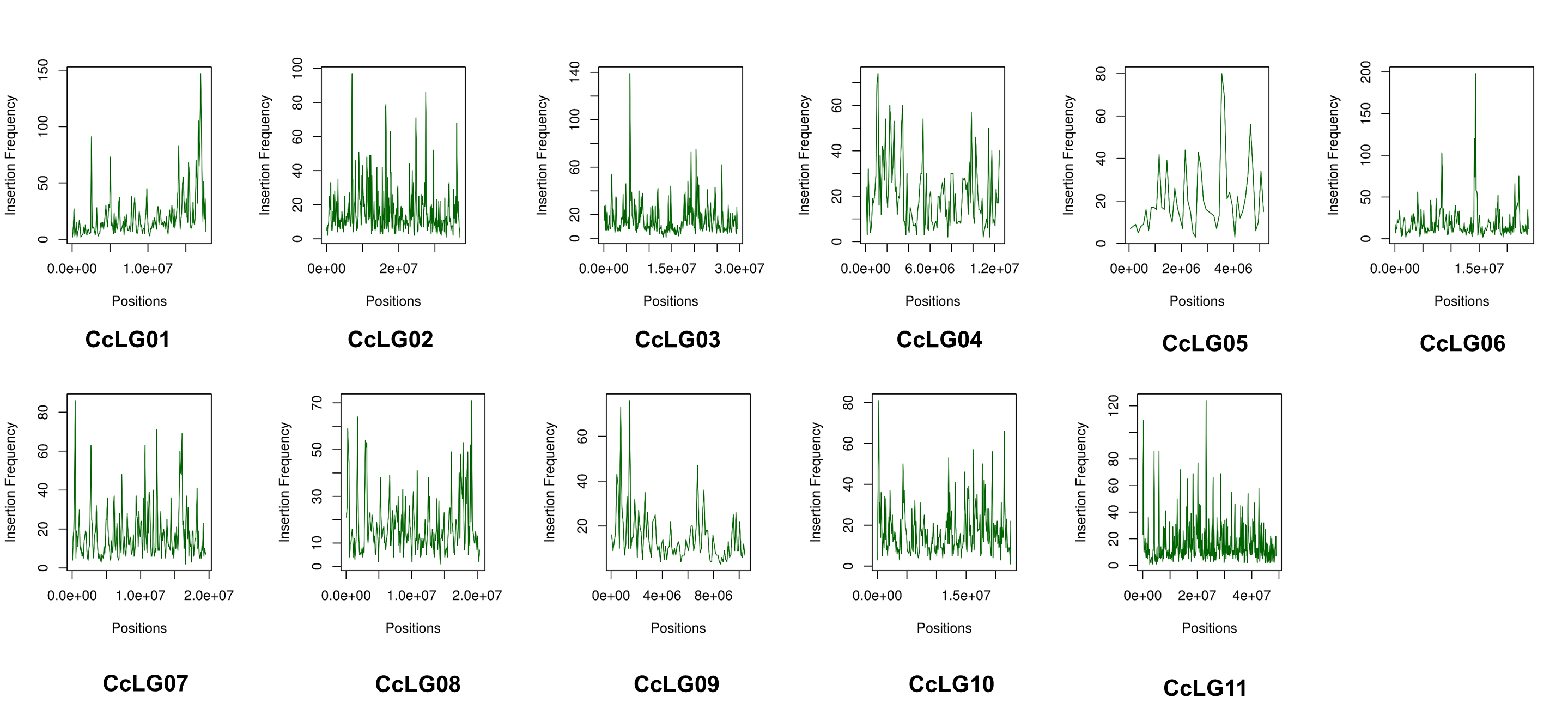


**Figure S3 Genome-wide identified insertion plots for all linkage groups**

Chromosomal distribution of genome-wide insertions identified after mapping of resistant parent (HTP), high trait bulk (HTB), and low trait bulk (LTB) on the reference genome. X-axis represents the positions of Indels into the genome and Y-axis represents the Indel frequency at particular position into the genome


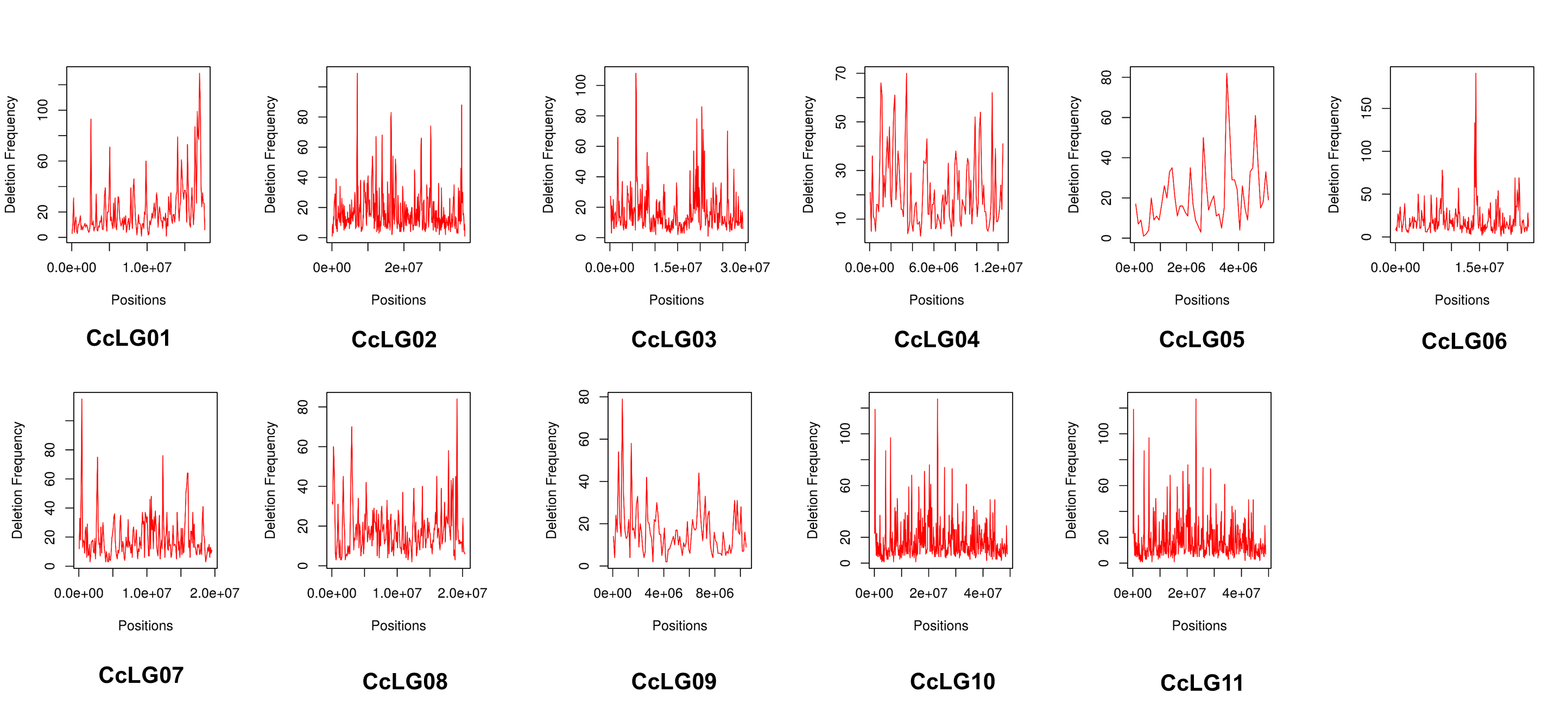


**Figure S4 Genome-wide identified deletion plots for all linkage groups**

Chromosomal distribution of genome-wide deletion identified after mapping of resistant parent (HTP), high trait bulk (HTB), and low trait bulk (LTB) on the reference genome. X-axis represents the positions of Indels into the genome and Y-axis represents the Indel frequency at particular position into the genome


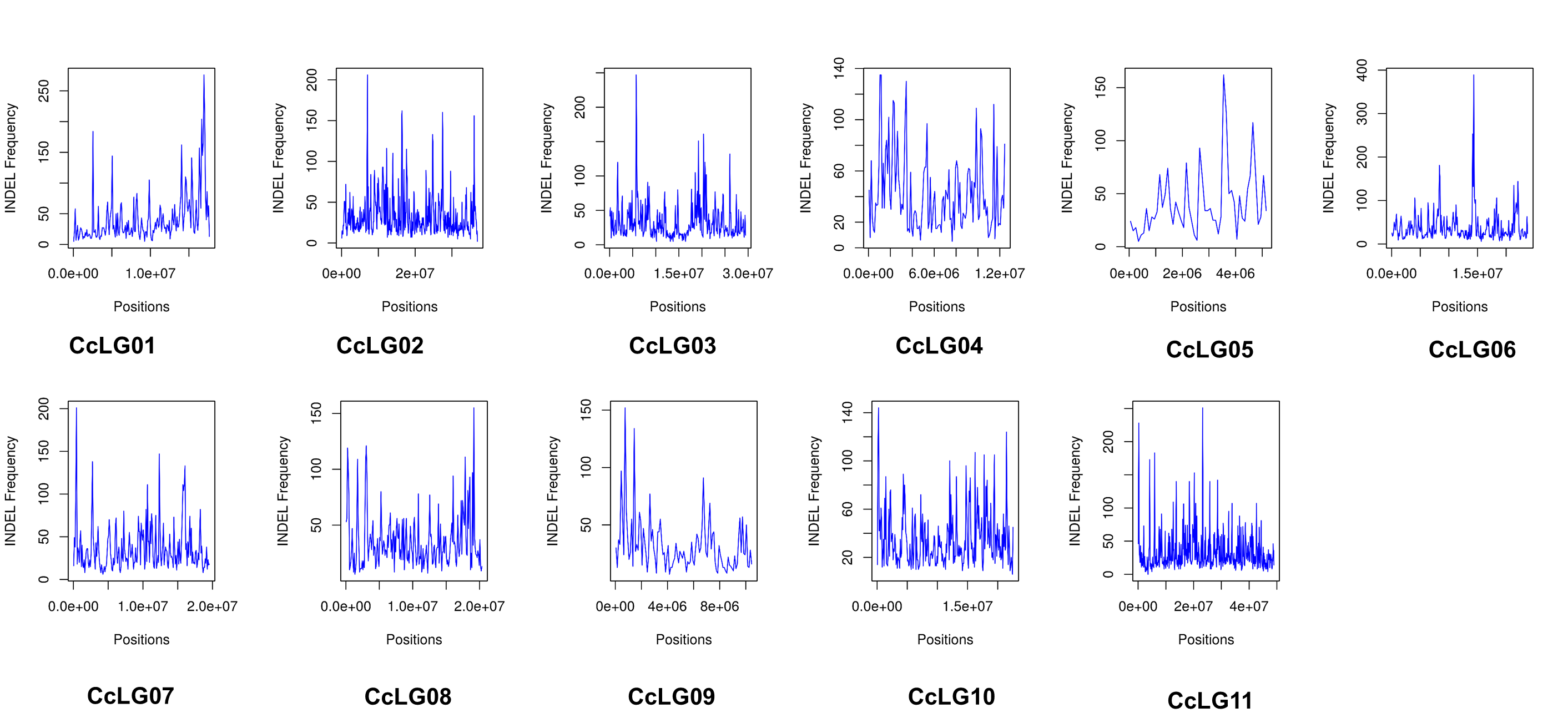


**Figure S5 Genome-wide identified Indels plots for all linkage groups**

Chromosomal distribution of genome-wide Indels identified after mapping of resistant parent (HTP), high trait bulk (HTB), and low trait bulk (LTB) on the reference genome. X-axis represents the positions of Indels into the genome and Y-axis represents the Indel frequency at particular position into the genome

**
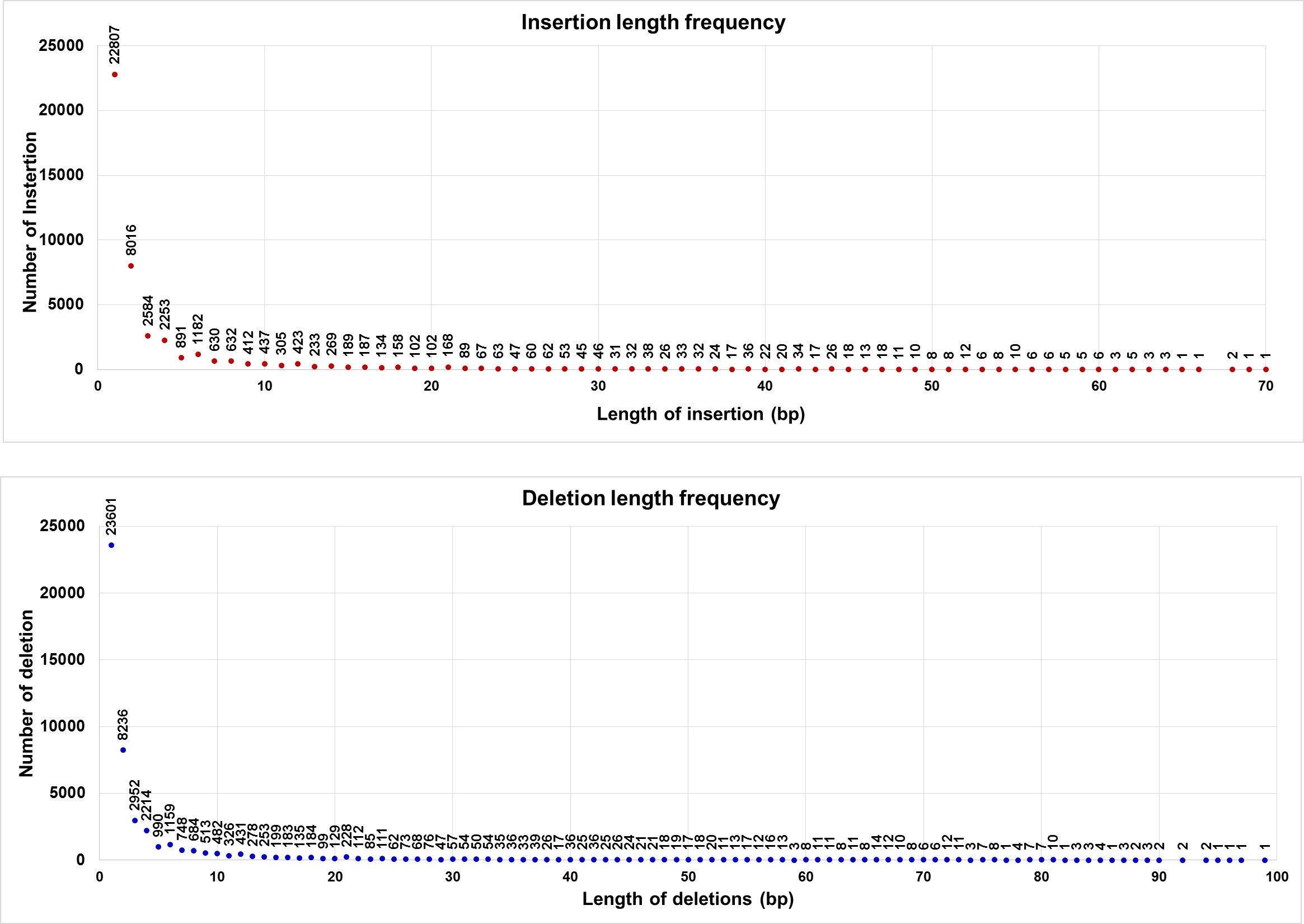
**

**Figure S6 Number and length of insertions and deletions identified after mapping**

A) Number and length of genome-wide insertions identified after mapping resistant parent (HTP), high trait bulk (HTB), and low trait bulk (LTB) on the reference genome. X-axis represents the length of insertions in bp and Y-axis represents the total number of insertions for a specific length. B) Number and length of genome-wide deletions identified after mapping of HTP, HTB, and LTB on the reference genome (RG). X-axis represents the length of deletions in bp and Y-axis represents the total number of deletions for a specific length.
